# Poor communication by health care professionals may lead to life-threatening complications: examples from two case reports

**DOI:** 10.12688/wellcomeopenres.15042.1

**Published:** 2019-01-22

**Authors:** Abhishek Tiwary, Ajwani Rimal, Buddhi Paudyal, Keshav Raj Sigdel, Buddha Basnyat

**Affiliations:** 1 Department of Internal Medicine, Patan Academy of Health Sciences, Lalitpur, Nepal; 2 Oxford University Clinical Research Unit, Patan hospital, Lalitpur, Nepal

**Keywords:** Communication, methotrexate, tuberculosis

## Abstract

We report two cases which highlight the fact how poor communication leads to dangerously poor health outcome. We present the case of a 50-year-old woman recently diagnosed with rheumatoid arthritis from Southern Nepal presented to Patan hospital with multiple episodes of vomiting and oral ulcers following the intake of methotrexate every day for 11 days, who was managed in the intensive care unit. Similarly, we present a 40-year-old man with ileo-caecal tuberculosis who was prescribed with anti-tubercular therapy (ATT) and prednisolone, who failed to take ATT due to poor communication and presented to Patan Hospital with features of disseminated tuberculosis following intake of 2 weeks of prednisolone alone. These were events that could have been easily prevented with proper communication skills. Improvement of communication between doctors and patients is paramount so that life-threatening events like these could be avoided.

## Introduction

Communication refers to exchanging information with the help of different mediums, such as speaking, writing or body language ^1^. It is of great importance in the field of medicine. Effective physician-patient communication is vital as it is related with favourable health outcomes such as increased patients satisfaction, compliance and overall health status ^2^. A study in 2008 by Bartlett G *et al.* concluded that communication problems with patients lead to increased preventable adverse effects which were mostly drug-related ^3^. It has been estimated that 27% of medical malpractice is the result of the communication failures. Better communication can reduce medical errors and patient injury ^4^. Poor communication can result in various negative outcomes, such as decreased adherence to treatment, patients dissatisfaction and inefficient use of resources ^5^. The cases discussed here highlight the importance of proper communication, how such unfortunate events could have been prevented with good communication skills. The traditional medical education curriculum in South Asia usually focuses more on technical expertise than teaching communication skills. This fact has hindered the capacity of technically expert health professionals to effectively communicate with their patients regarding the disease and treatment approach ^6, 7^. Thus, a concerted effort needs to be made to improve the communication skills of health professionals in South Asia.

## Case reports

### Case 1

A 50-year-old woman diagnosed with rheumatoid arthritis (RA) 3 weeks previously presented to Emergency Department of Patan Hospital in June of 2018 with complaints of multiple episodes of vomiting and oral ulcers for 5 days. She had a history of multiple joint pain for a year, for which she sought medical attention in New Delhi, India as her son used to work there. She visited New Delhi with her neighbour, and there was diagnosed with RA. As per the standard treatment of RA, her treating rheumatologist prescribed her 15 mg methotrexate once weekly and 5 mg folic acid twice weekly without emphasizing that methotrexate is to be taken weekly and not daily. The pharmacist also failed to stress the weekly dose schedule. Unfortunately, she consumed methotrexate 15 mg daily for 11 days. At 11th day, she presented with those above complaints to the National Medical College and Teaching Hospital near her home in Birgunj, in the southern plains of Nepal. There she was managed conservatively with folic acid and fluids for 2 days, then referred to our centre for further management. She had ongoing vomiting and her examination of the oral cavity revealed multiple erythematous and ulcerative lesions. Her total white blood cell count (WBC) was 2400/µl (normal range, 4000–11000/µl), with an absolute neutrophil count (ANC) of 1200/µl (normal range, 1500–8000/µl), haemoglobin of 9 g/dl (12–15 g/dl) and platelets of 84000/µl (150,000–450,000/µl). She was immediately admitted to the intensive care unit (ICU) for methotrexate toxicity (myelosuppression and mucositis). Her methotrexate was stopped and she was managed with leucovorin (15 mg once daily), GM-CSF (300 µg once daily) and nasogastric feeding as she was unable to eat anything because of the oral ulcers.

After 3 days in the ICU, she was transferred to the ward, where treatment with leucovorin and GM-CSF was continued at the same dose. She was discharged after a total of 11 days of hospital stay when her blood counts came back to within the normal range (WBC, 12300/µl; ANC, 6888/µl). Her haemoglobin increased to 13 g/dl and her platelet reached 340,000/µl. Her oral lesions subsided, and she was able to feed orally. She was started back on the correct dosage of methotrexate (15 mg once weekly) and counselled about the disease, medications (dosage and adverse effects) and was advised to follow up in rheumatology clinic. She has been followed-up every 3 months since then, is in remission and is taking medications properly.

### Case 2

A 40-year-old man from hills of Nepal presented to the emergency department of Patan Hospital in August 2018 with complaints of weakness in the right half of the body, deviation of the left side of the face and slurring of speech for 4 days. At 3 weeks prior to this, he had visited another tertiary level hospital in Kathmandu for pain in the lower abdomen and fever, where he was diagnosed as having ileo-cecal tuberculosis based on colonoscopy and biopsy with positive Ziehl-Neelson staining. He was then prescribed with antitubercular therapy (ATT) that included 3 tablets of Fixed dose combination consisting of isoniazid 75 mg, rifampicin 150 mg, pyrazinamide 400 mg and ethambutol 275 mg once daily and prednisolone 40 mg once daily. He was advised to take ATT from a health centre near his residence, whereas prednisolone was dispensed from the hospital pharmacy. Unfortunately, he just took prednisolone, but no ATT. As a result, he ended up in emergency with the aforementioned complaints. On evaluation, his chest x-ray showed features of pulmonary tuberculosis. Cerebral spinal Fluid (CSF) analysis was done which showed red blood cells (RBC) 200/µl (normal value, 0/µl), WBC 64/µl (normal range, 0–5/ µl), neutrophil 24%, lymphocytes 64%, protein 294 mg/dl (normal range, 15–45 mg/dl) and sugar 49 mg/dl (normal range, 50–80 mg/dl). Cerebrospinal fluid GeneXpert testing was positive for *Mycobacterium tuberculosis*. He was then diagnosed as disseminated tuberculosis with meningeal involvement and was admitted to Patan Hospital with ATT (3 tablets of fixed-dose combination consisting of Isoniazid 75mg, Rifampicin 150 mg, Pyrazinamide 400 mg and Ethambutol 275mg once daily) and dexamethasone (6 mg three times a day) for 3 days. He was then discharged with ATT (same dose as above) and prednisolone (40 mg once daily) after proper counselling about the nature of the disease and site of availability of anti-tubercular drugs. He came in for follow-up after 2 weeks with improvement in the symptoms and has been taking all medications properly. 

## Discussion

In the discussed cases, the treating physicians had used the standard treatment protocol to best serve their patients. They used their medical knowledge in an appropriate manner to treat the disease condition, but proper communication with clear-cut emphasis on how and when to take the therapy, which is of utmost importance in achieving an overall positive health impact, was lacking. Had the doctors properly counselled and educated the patients regarding the disease, treatment options and the correct way of taking medications, these mishaps could have been prevented. Another major part of the communication involves the judgment of the doctor in figuring out how much the patient understood. As our patients were not literate, they could have explained about the disease and especially the weekly dosing of methotrexate and the availability and importance of ATT very clearly to the patient family. In South Asian countries like Nepal, the patient seldom is alone and therefore making things clear to the patient’s family is obviously a very important option that needs to be utilized to improve communication against the background of rampant illiteracy. In Nepal, only 48.6% of the population is literate; hence this fact needs to be kept in mind when explaining about diseases and prescribing drugs, especially regarding medicines that have dangerous side-effects ^8^.

In Nepal, 25.2% population fall below the poverty line and 3.2% population are unemployed ^9^. The young working generation have to leave their house for better employment opportunities, meaning they aren’t able to take care of their parents. In one of our cases, the son had to work in India for better employment opportunities and the patient came with her neighbour with whom the treating physician did not spend any time. It is possible that if the son had been there, he may well have been more concerned and asked more questions to the doctor. However, it is the responsibility of the health care professional to try to make sure the patient and their family have understood the matter clearly. There was also no caution mentioned by the pharmacy where the patient bought the medicine explaining the weekly (and not daily) dosing schedule of methotrexate. Hence there was failure of clear communication at various levels that led to this mishap.

Problems in doctor‐patient communication have received little attention as a potential but a remediable cause of health hazards, especially in a setting like this one in South Asia. Communication during the medical interaction among the health practitioner and the patient has a pivotal role in creating a positive health impact that includes drug adherence, future decision making on the interventions and modifying the health behaviours of the patient. We consider the cost and the negative impact on the outcome of the health from poor communication, which includes non-adherence to drugs regimens that will increase the burden of the cost of the total drug therapy, poor health outcomes, and unnecessary treatment and investigations. Different measures need to be considered to improve the communication between doctors and patients which would improve the overall health outcome. The measures include providing communication skills training to health care professionals and regular evaluation of communication skills of these professionals by interviewing the patients after a consultation.

## Conclusion

Clear communication is vital in the proper treatment of the patient especially against the background of rampant illiteracy in countries such as Nepal in South Asia. Poor Communication may lead to life-threatening complications, as in our patients. For better communication practice, proper communication training to health care professionals including pharmacists is paramount.

## Consent

Informed consent for publication of their clinical details, in the form of a fingerprint, was obtained from the patients.

## Data availability

All data underlying the results are available as part of the article and no additional source data are required.
