# Supplementary material for: Poor communication by health care professionals may lead to life-threatening complications: examples from two case reports
Source: Wellcome Open Res. 2019 Jan 22;4:7. [Version 1] doi: 10.12688/wellcomeopenres.15042.1 (PMC6694717; doi:10.12688/wellcomeopenres.15042.1)
Supplement: Peer review history

# Peer review: Reviewer response for version 1

**DOI:** 10.21956/wellcomeopenres.16411.r36091

**Published:** 2019-08-12

**Authors:** Jill Allison

**Affiliations:** 1 Division of Community Health and Humanities, Faculty of Medicine, Memorial University of Newfoundland, St. John's, NL, Canada

- This article provides two cases where a lack of information and clear understanding of prescription medication contributed to morbidity and unnecessary suffering for the patients. The cases are linked to a lack of health professional engagement with the patient and failure to ensure full understanding of medication instructions. The cases and events surrounding are clearly described. The outcomes are also clearly described.
- The clinical scenario is well described but it would be helpful to know what steps were taken with these two patients to prevent similar circumstances. There is no mention of what was done to educate and inform the patient or their families on discharge. Was there an interdisciplinary team involved to try to ensure the patient got sufficient information and how was their level of comprehension assessed?
- There is a bit of repetition in the discussion and not many concrete suggestions for improving the skills of physicians in this area. Continuing medical education? Cultural competency teaching?
- There are a few grammatical errors that could be corrected to improve the paper. 
- Overall, an important concept for discussion and excellent examples of why the discussion must happen. 

I confirm that I have read this submission and believe that I have an appropriate level of expertise to confirm that it is of an acceptable scientific standard.

# Peer review: Reviewer response for version 1

**DOI:** 10.21956/wellcomeopenres.16411.r35794

**Published:** 2019-07-02

**Authors:** Sharad Onta

**Affiliations:** 1 Department of Community Medicine and Public Health, Institute of Medicine, Tribhuvan University, Kathmandu, Nepal; 2 Nepal Public Health Foundation, Kathmandu, Nepal

**Introduction:**

- This section should focus on contextual facts about the central issue of the manuscript, communication in clinical practice in the present context. It is better to avoid assessment of the contents of the cases and conclusion with recommendation. 
- The statement “ *The cases discussed here highlight the importance of proper communication, how such unfortunate events could have been prevented with good communication skills”* indicates to the assessment of upcoming contents of the manuscript. It seems inappropriate in the introduction. (It better fits in the discussion).
- The last phrase of this section “ *Thus, a concerted effort needs to be made to improve the communication skills of health professionals in South Asia”* carries a notion of recommendation, which seems premature for this section of the manuscript. (It can be moved to the conclusion).
- It will be better to highlight the objective and rationale of presenting cases in this section. It provides the space for the authors to justify importance of communication in clinical practice.

**Cases:**

- Adequate exploration of the facts as the evidence of poor communication in health service/clinical practices and highlights of these facts (findings) are necessary in presentation of the cases for justification of explanations narrated in the section of discussion. The cases in the manuscript look weak, as the communication aspects are not adequately elaborated on. Elaboration of communication dimension in the case presentation is desirable and, hence, suggested.
- As emphasized in the discussion section, and in the conclusion, of the manuscript, socio-economic characteristics of the service seekers are not clearly mentioned in the cases. Therefore, rationalization of importance of communication in the basis of these attributes is not well justifiable.  

 

**Discussion:**

- The discussion should further attempt to establish credible links between doctor-patient communication and implications seen in the discussed cases. Other potential causes for such implications should be excluded to the best possible extent.
  Few examples:
- In case 1 – it should be explored in depth whether the attitude and faith of patients to recover earlier by getting medicine in more (frequently) quantity than prescribed dose could be the reason for this situation.
- In case 2 – role of poor communication is not established clearly. Other possible reasons for not taking ATT like unavailability of medicines, distance to the health centres, and so on should be excluded to establish the role of communication. If prednisolone was the underlying cause of complication of the case, it should be analyzed, whether dispensing prednisolone alone without AT medicines to the patient was a right practice/protocol and correlate with the communication.

 

**Confidentiality:**

- In case 1 – name of the referring hospital as *National Medical College and Teaching Hospital* is mentioned whereas in the case 2 – it is mentioned as *another tertiary level hospital in Kathmandu*. It is better to maintain the consistency.

  

**Conclusion:**

- The conclusion is not well based in facts of cases. The manuscript has justified the importance of communication (in Nepal) in the background of *rampant illiteracy*. However, literacy and other socio-economic status of the patients in both cases are not known.

 

**General:**

- Language could be improved.
- Manuscript has addressed very relevant and useful issues. It should be considered for indexing after improvement incorporating all comments. 

     

I confirm that I have read this submission and believe that I have an appropriate level of expertise to confirm that it is of an acceptable scientific standard.
